# Exploratory profiles of phenols, parabens, and per- and poly-fluoroalkyl substances among NHANES study participants in association with previous cancer diagnoses

**DOI:** 10.1038/s41370-023-00601-6

**Published:** 2023-09-18

**Authors:** Amber L. Cathey, Vy K. Nguyen, Justin A. Colacino, Tracey J. Woodruff, Peggy Reynolds, Max T. Aung

**Affiliations:** 1 Department of Environmental Health Sciences, University of Michigan, School of Public Health, Ann Arbor, MI USA; 2 Department of Biomedical Informatics, Harvard Medical School, Boston, MA USA; 3 Program on Reproductive Health and the Environment, Department of Obstetrics, Gynecology, and Reproductive Sciences, University of California, San Francisco, San Francisco, CA USA; 4 Department of Epidemiology and Biostatistics, University of California, San Francisco, San Francisco, CA USA; 5 Department of Population and Public Health Sciences, University of Southern California, Keck School of Medicine, Los Angeles, CA USA

## Abstract

**Background:**

Some hormonally active cancers have low survival rates, but a large proportion of their incidence remains unexplained. Endocrine disrupting chemicals may affect hormone pathways in the pathology of these cancers.

**Objective:**

To evaluate cross-sectional associations between per- and polyfluoroalkyl substances (PFAS), phenols, and parabens and self-reported previous cancer diagnoses in the National Health and Nutrition Examination Survey (NHANES).

**Methods:**

We extracted concentrations of 7 PFAS and 12 phenols/parabens and self-reported diagnoses of melanoma and cancers of the thyroid, breast, ovary, uterus, and prostate in men and women (≥20 years). Associations between previous cancer diagnoses and an interquartile range increase in exposure biomarkers were evaluated using logistic regression models adjusted for key covariates. We conceptualized race as social construct proxy of structural social factors and examined associations in non-Hispanic Black, Mexican American, and other Hispanic participants separately compared to White participants.

**Results:**

Previous melanoma in women was associated with higher PFDE (OR:2.07, 95% CI: 1.25, 3.43), PFNA (OR:1.72, 95% CI: 1.09, 2.73), PFUA (OR:1.76, 95% CI: 1.07, 2.89), BP3 (OR: 1.81, 95% CI: 1.10, 2.96), DCP25 (OR: 2.41, 95% CI: 1.22, 4.76), and DCP24 (OR: 1.85, 95% CI: 1.05, 3.26). Previous ovarian cancer was associated with higher DCP25 (OR: 2.80, 95% CI: 1.08, 7.27), BPA (OR: 1.93, 95% CI: 1.11, 3.35) and BP3 (OR: 1.76, 95% CI: 1.00, 3.09). Previous uterine cancer was associated with increased PFNA (OR: 1.55, 95% CI: 1.03, 2.34), while higher ethyl paraben was inversely associated (OR: 0.31, 95% CI: 0.12, 0.85). Various PFAS were associated with previous ovarian and uterine cancers in White women, while MPAH or BPF was associated with previous breast cancer among non-White women.

**Impact Statement:**

Biomarkers across all exposure categories (phenols, parabens, and per- and poly- fluoroalkyl substances) were cross-sectionally associated with increased odds of previous melanoma diagnoses in women, and increased odds of previous ovarian cancer was associated with several phenols and parabens. Some associations differed by racial group, which is particularly impactful given the established racial disparities in distributions of exposure to these chemicals. This is the first epidemiological study to investigate exposure to phenols in relation to previous cancer diagnoses, and the first NHANES study to explore racial/ethnic disparities in associations between environmental phenol, paraben, and PFAS exposures and historical cancer diagnosis.

## Introduction

Prostate and breast cancer are the most commonly diagnosed cancers among men and women, respectively [1]. Despite their prevalence, risk factors explaining the majority of cases remain elusive [2]. Previous work has shown that genetic heritability does not fully explain the incidence and outcomes of these cancers, thus multiple environmental and social factors are likely to be involved in the initiation and progression of these diseases [3]. Prostate and breast cancer are both hormone-mediated cancers, as are other less common cancer types including ovarian cancer, endometrial cancer, testicular cancer, thyroid cancer, and melanoma. Growth and progression of these cancer types depend largely on endogenous steroid and thyroid hormones [4], therefore identifying environmental insults that impact these hormone levels may be important for discovery of new cancer prevention and mitigation methods. These efforts could include targeted environmental health interventions to reduce exposure to these chemicals in high-risk individuals or cancer patients, regulations to limit the exposure of these chemicals in the general population, and the replacement of these chemicals with safer alternatives.

Many environmental toxicants have been identified as endocrine disruptors, including phenols, parabens, and per- and polyfluoroalkyl substances (PFAS). Human exposure to phenols and parabens occurs most commonly via plastic food/beverage packaging and personal care products. PFAS chemicals are found in stain resistant fabrics and flame retardant furniture, are persistent in the environment, and can bioaccumulate inside the body following exposure. Previous work has shown these chemicals to have effects on circulating concentrations of estrogens [5, 6], thyroid hormones [6–8], and testosterone [6, 9] in human studies. Further, effects on hormones have been identified as a key characteristic of carcinogenesis [10]. Despite the established endocrine disrupting potential of these chemicals, few epidemiology studies have assessed their relationships with endocrine-active cancer outcomes. Several case control studies have shown positive or suggestive associations between breast cancer and bisphenol-A (BPA) [11] and PFAS chemicals [12–15], but similar studies involving other emerging phenols or other cancer types are lacking.

The National Health and Nutrition Examination Survey (NHANES) is a United States nation-wide biomonitoring effort which has demonstrated evidence of widespread human exposure to environmental toxicants including phenols, parabens, and PFAS [16]. NHANES also provides self-reported cancer diagnoses for all participating individuals aged 20 years and older, constituting an ideal dataset for conducting preliminary analyses to evaluate the relationships between environmental chemicals and cancer outcomes. Therefore, the aim of this study was to utilize NHANES data from 2005 to 2018 to conduct a cross-sectional study evaluating associations between current exposure levels to phenols, parabens, and PFAS chemicals and previous endocrine-active cancer diagnoses. The results from this study can help identify the potential role of environmental toxicants in prospective studies of cancer.

## Methods

### Study population

**Fig. 1:**
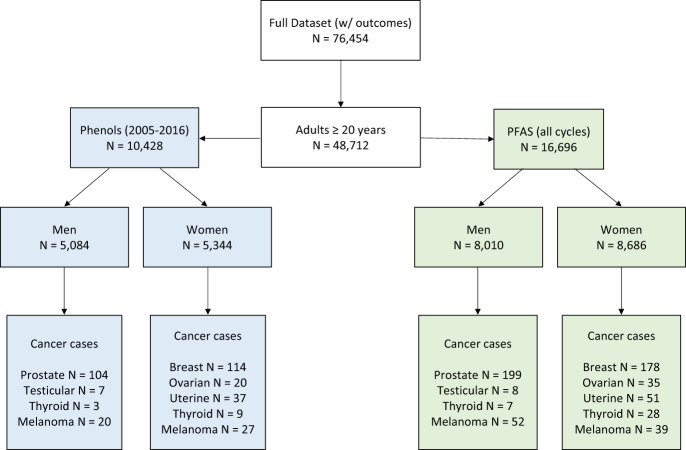
Flow chart for building the final analytical datasets. Sample size are indicated in green boxes for PFAS chemicals and blue boxes for phenols/parabens.

Data from NHANES was used for the present analysis. NHANES is composed of a non-institutionalized, nationally representative, sample of children and adults and is used to assess the health and nutritional status of the United States population. A flowchart depicting how we built our analytical datasets is presented in Fig. 1. From NHANES data collected between 2005 and 2018, we extracted demographic variables, self-reported cancer diagnoses from the medical conditions questionnaire, and concurrent biomarker concentrations of phenols, parabens, and PFAS. We first restricted our dataset to all individuals 20 years and older with complete data on selected covariates (age, serum concentrations of the tobacco smoke metabolite cotinine, poverty-income ratio, race, education, body mass index, and creatinine (for phenol/paraben analysis only)) for an initial sample size of 48,712 people. Additionally, in NHANES there are non-overlapping participants with measurements for different exposure chemical panels, thus, we created two separate analytical datasets – one for phenols/parabens and one for PFAS. After removing individuals missing biomarker data, the PFAS dataset contained 16,696 people and the phenols/parabens dataset contained 10,428 people (phenols and parabens were not measured in the 2017–2018 cycle). Our study goal was to focus on sex-specific relationships between environmental PFAS, phenols and parabens exposure with previous cancer diagnosis, partly due to sexual dimorphic profiles for cancer risk. Therefore, to evaluate sex-specific cancers, both datasets were separated between males and females for final sample sizes of 8010 men and 8686 women in the PFAS analysis and 5084 men and 5344 women in the phenol/paraben analysis.

### Biomarker assessment

We included NHANES measures of a total of seven PFAS chemicals and 12 phenols/parabens. Five PFAS chemicals were measured in all cycles from 2005 to 2018: perfluorohexane sulfonic acid (PFHS), 2-(N-methyl-PFOSA)acetic acid (MPAH), perfluorodecanoic acid (PFDE), perfluorononanoic acid (PFNA), and perfluoroundecanoic acid (PFUA). Perfluorooctanoic acid (PFOA) and perfluorooctane sulfonic acid (PFOS) were included from all cycles except 2013–2014. PFAS were quantified from serum samples using high performance liquid chromatography-turbo ion spray ionization-tandem mass spectrometry. Seven phenols/parabens were measured in all cycles between 2005 and 2016: bisphenol-A (BPA), benzophenone-3 (BP3), triclosan (TCS), methyl paraben (MPB), ethyl paraben (EPB), propyl paraben (PPB), and butyl paraben (BPB). Five additional phenols/parabens were measured from 2013 to 2016: bisphenol-F (BPF), bisphenol-S (BPS), triclocarban (TCC), 2,4-dichlorophenol (DCP24), and 2,5-dichlorophenol (DCP25). Phenols and parabens were quantified in urine samples using on-line solid phase extraction coupled to high performance liquid chromatography and tandem mass spectrometry.

### Cancer outcome assessment

Self-reported cancer diagnoses were obtained from the medical conditions questionnaire administered to all participants 20 years and older. Participants were first asked “Have you ever been told by a doctor or other health professional that you had cancer or a malignancy of any kind?” Those who responded yes were then asked to indicate which type of cancer it was, and they were able to indicate up to three different cancer types. We extracted data for 7 cancer types: breast, ovarian, uterine, prostate, testicular, thyroid, and melanoma. We also created a variable for combined reproductive cancers which included breast, ovarian, and uterine cancers for women and prostate and testicular cancer for men. Testicular cancer and thyroid cancer among men were excluded from regression analyses because of low case numbers (*N* < 10).

### Statistical analyses

Demographic characteristics among participants 20 years and older who provided data on at least one exposure-outcome pair were tabulated for both PFAS and phenol/paraben populations. Case/control sample sizes of cancer outcomes were tabulated, and distributions of all exposure variables were evaluated. All exposure biomarkers were right-skewed and were thus natural log-transformed for all analyses. Concentrations of BPB, EPB, and triclocarban were measured below the limit of detection (LOD) in more than 50% of samples, so these were treated as categorical variables with all concentrations below the LOD as the reference group and the remaining concentrations split between those below the median and those at or above the median of detectable values. All concentrations below the LOD were imputed with the LOD divided by the square root of two. Associations between exposure biomarkers and cancer outcomes were estimated using logistic regression. Based on the literature of potential cancer risk factors [17] we considered demographic, social, and biological covariates to determine potential confounders, and we examined bivariate associations between exposures, outcome, and these potential confounders to build our adjusted models. Based on these relationships, adjusted models included age at the time of survey, natural log-transformed cotinine, poverty-income ratio, race, education, body mass index, and an indicator variable for NHANES cycle to capture changing exposure and outcome trends over time. Phenol/paraben models also were adjusted for natural log-transformed urinary creatinine to account for differences in urinary dilution. We also considered adjusting melanoma models for self-reported sunscreen use due to many brands being a source of phenol exposure [18], but there was not enough overlap in participants who provided sunscreen use data and those who reported having melanoma. All results are presented as the odds of previous cancer diagnosis with an interquartile range (IQR) increase in current exposure biomarker concentration. All analyses were conducted in R version 4.0.4.

### Effect modification by race

To explore the possibility of race as a social construct proxy of structural social factors and an effect modifier on the associations between environmental exposures and cancer outcomes, we ran sensitivity analyses in which effect estimates were calculated among non-White racial groups separately (non-Hispanic Black, Mexican American, and other Hispanic) and compared to effects among White participants only. Models included interaction terms specific to each White/non-White pair such that the p-value of the interaction term (p-int) could be interpreted as the significance of the difference between those two groups (*p* < 0.05 was considered statistically significant).

### Sampling weights sensitivity analyses

As a sensitivity analysis, we accounted for survey sampling weights in all adjusted models to determine consistency in findings in unweighted analyses. Application of the survey weights accounts for sampling based on demographic factors and produces estimates that are representative of the non-institutionalized general US population. Across multiple NHANES cycles, we utilized R to apply the weighting algorithm explained by Nguyen and colleagues [16], which prioritizes weights on the smallest subsample of biomarker data for integration of weights.

## Results

Demographic information is shown in Table 1. Both PFAS and phenol/paraben populations had similar proportions of men and women, with slightly more women. Across both sexes and datasets, the median age was about 49 years, the median poverty income ratio was about 2, and the median body mass index was about 27 kg/m^2^. Serum cotinine concentrations were higher in men than in women. Racial distributions were consistent between sex and chemical datasets, with approximately 40% non-Hispanic White, 20% non-Hispanic Black, 15% Mexican American, 10% other Hispanic, and 15% other. The majority of participants reported having an education level of at least some college.

**Table 1:** Demographic characteristics of NHANES participants, 20 years and older, who provided data on at least one PFAS or phenol/paraben chemical.

	PFAS Population		Phenol Population	
	Men (*N* = 8010)	Women (*N* = 8686)	Men (*N* = 5084)	Women (*N* = 5344)
**Median (SD)**				
**Age**	50 (17.9)	49 (17.8)	49 (17.8)	48 (17.9)
**Poverty Income Ratio**	2.19 (1.63)	1.99 (1.62)	2.2 (1.64)	1.98 (1.64)
**Serum Cotinine**	0.063 (140)	0.03 (108)	0.75 (138)	0.034 (118)
**BMI**	27.4 (5.62)	27.4 (7.34)	27.4 (5.77)	27.1 (6.89)
***N*** **(%)**				
**Race**				
Non-Hispanic White	3358 (41.9%)	3428 (39.5%)	2206 (43.4%)	2232 (41.8%)
Non-Hispanic Black	1693 (21.1%)	1867 (21.5%)	1106 (21.8%)	1179 (22.1%)
Mexican American	1212 (15.1%)	1370 (15.8%)	792 (15.6%)	844 (15.8%)
Other Hispanic	731 (9.1%)	922 (10.6%)	447 (8.8%)	548 (10.3%)
Other	1016 (12.7%)	1099 (12.7%)	533 (10.5)	541 (10.1%)
**Annual Household Income**				
Less than $20k	1353 (18.9%)	1751 (22.5%)	915 (20.0%)	1169 (24.2%)
[$20k–$45k)	2252 (31.5%)	2449 (31.5%)	1416 (30.9%)	1516 (31.3%)
[$45k–$75k)	1449 (20.3%)	1541 (19.8%)	947 (20.7%)	925 (19.1%)
$75 K or more	2089 (29.2%)	2027 (26.1%)	1306 (28.5%)	1228 (25.4%)
**Education**				
Less than 9th grade	856 (10.7%)	881 (10.2%)	583 (11.5%)	549 (10.3%)
9th–11th grade	1204 (15.1%)	1145 (13.2%)	770 (15.2%)	784 (14.7%)
Diploma or equivalent	1848 (23.1%)	1857 (21.4%)	1203 (23.7%)	1203 (22.5%)
Some college/AA	2217 (27.7%)	2810 (32.4%)	1354 (26.7%)	1626 (30.5%)
College grad or more	1874 (23.4%)	1977 (22.8%)	1170 (23.0%)	1174 (22.0%)

*SD* Standard deviation, *BMI* Body mass index.

Distributions of biomarker concentrations are shown in Supplementary Tables 1 and 2, and counts of cancer diagnoses and controls are shown in Table 2. Prostate cancer was the most frequently reported malignancy among men (PFAS subset *N* = 199; phenol/paraben subset *N* = 104) and breast cancer was the most frequently reported among women (PFAS *N* = 178; phenol/paraben *N* = 114).

**Table 2:** Cancer case numbers, among those who provided complete data on selected covariates and cancer outcome data, between the PFAS and phenols analytical datasets.

		PFAS Population		Phenols Population	
	*N* (%)	Men (*N* = 6360)	Women (N = 6886)	Men (*N* = 3606)	Women (N = 3807)
All Reproductive Cancers*	Yes	207 (3.3%)	255 (3.7%)	111 (3.1%)	168 (4.4%)
	No	6153 (96.7%)	6631 (96.3%)	3495 (96.9%)	3639 (95.6%)
**Prostate Cancer**	Yes	199 (3.1%)		104 (2.9%)	
	No	6161 (96.9%)		3502 (97.1%)	
**Testicular Cancer**	Yes	8 (0.1%)		7 (0.2%)	
	No	6352 (99.9%)		3599 (99.8%)	
**Breast Cancer**	Yes		178 (2.6%)		114 (3.0%)
	No		6708 (97.4%)		3693 (97.0%)
**Ovarian Cancer**	Yes		35 (0.5%)		20 (0.5%)
	No		6851 (99.5%)		3787 (99.5%)
**Uterine Cancer**	Yes		51 (0.8%)		37 (1.1%)
	No		6215 (99.2%)		3432 (98.9%)
**Melanoma**	Yes	52 (0.8%)	39 (0.6%)	20 (0.6%)	27 (0.7%)
	No	6308 (99.2%)	6847 (99.4%)	3586 (99.4%)	3780 (99.3%)
**Thyroid Cancer**	Yes	7 (0.1%)	28 (0.4%)	3 (0.1%)	9 (0.2%)
	No	6353 (99.9%)	6858 (99.6%)	3603 (99.9%)	3798 (99.8%)

^*^All reproductive cancers include prostate and testicular cancers for men and breast, ovarian, and uterine cancers for women.

**Fig. 2:**
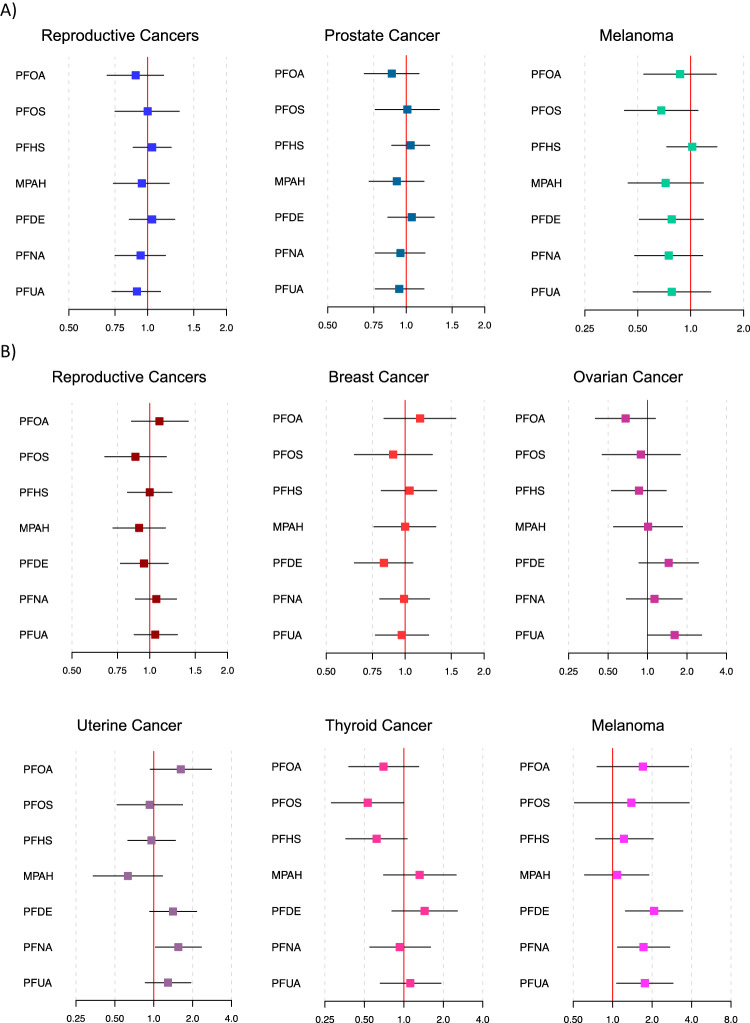
Odds of each cancer type with an IQR increase in each PFAS chemical. Effect estimates and 95% confidence intervals are reported among men (Panel **A**) and women (Panel **B**). Models adjust for age at the time of survey, cotinine, poverty-income ratio, race, education, body mass index, and an indicator variable for NHANES cycle.

Associations between current PFAS concentrations and odds of having a previous cancer diagnosis are depicted in Fig. 2 (corresponding numeric data is shown in Supplementary Table 3; numerical data with survey weights applied are shown in Supplementary Table 5). We did not observe any associations between PFAS biomarkers and previous cancer diagnoses in men. However, we did observe positive associations between several PFAS biomarkers and odds of previous melanoma among women. IQR increases in PFDE, PFNA, and PFUA were associated with 2.07 (95% CI: 1.25, 3.43), 1.72 (95% CI: 1.09, 2.73), and 1.76 (95% CI: 1.07, 2.89) times greater odds of previous melanoma diagnosis in women. There was also a positive association between an IQR increase in PFNA and odds of previous uterine cancer (OR: 1.55, 95% CI: 1.03, 2.34) and a marginally (0.5 ≤ p < 0.1) positive association between an IQR increase in PFUA and odds of previous ovarian cancer (OR: 1.61, 95% CI: 1.00, 2.59).

**Fig. 3:**
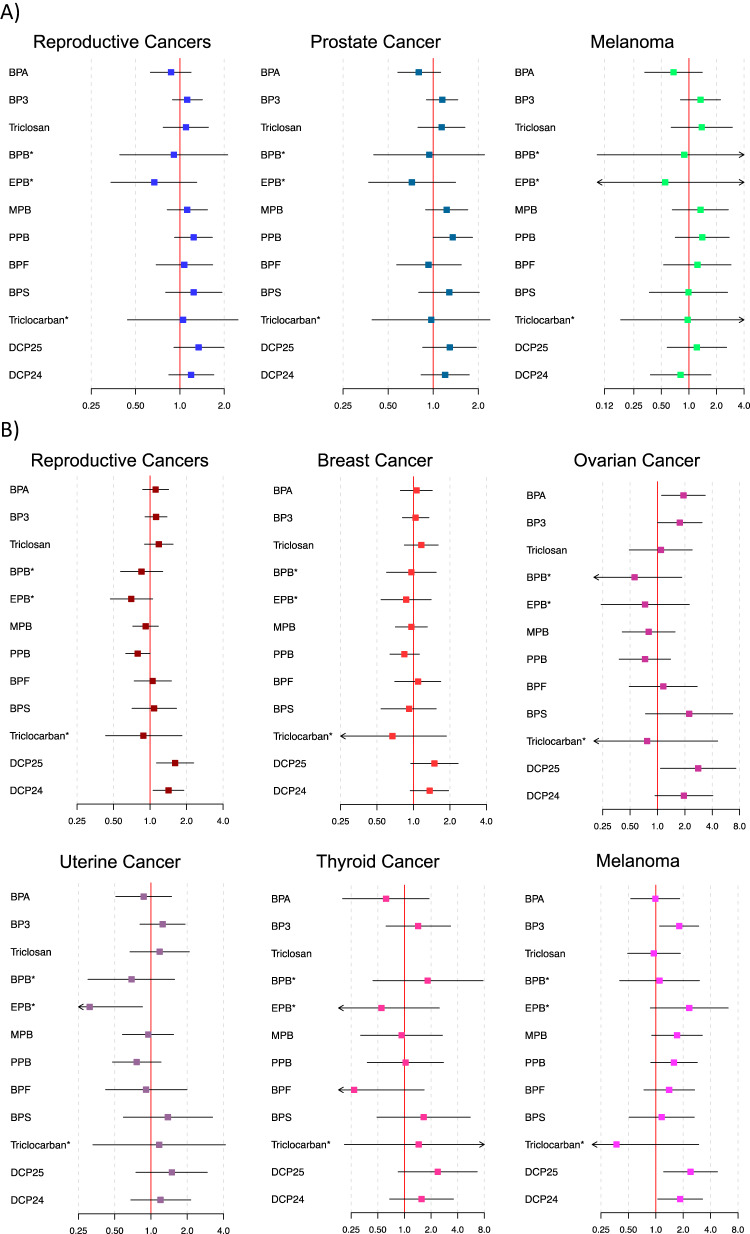
Odds of each cancer type with an IQR increase in each phenol/paraben chemical. Effect estimates and 95% confidence intervals are reported among men (Panel **A**) and women (Panel **B**). Models adjust for age at the time of survey, cotinine, poverty-income ratio, race, education, body mass index, an indicator variable for NHANES cycle, and creatinine.

Associations between current phenol/paraben concentrations and odds of having a previous cancer diagnosis are depicted in Fig. 3 (corresponding numeric data is shown in Supplementary Table 4; numerical data with survey weights applied are shown in Supplementary Table 6). There was a marginally (0.5 ≤ p < 0.1) positive association between an IQR increase in PPB and odds of previous prostate cancer diagnosis (OR: 1.35, 95% CI: 1.00, 1.83). Increased odds of previous reproductive cancer diagnosis among women was associated with IQR increases in DCP25 (OR: 1.61, 95% CI: 1.13, 2.29) and DCP24 (OR: 1.42, 95% CI: 1.06, 1.90). These findings were likely driven by positive associations with both previous breast cancer diagnosis (DCP25 OR: 1.49, 95% CI: 0.95, 2.34; DCP24 OR: 1.36, 95% CI: 0.94, 1.95) and previous ovarian cancer diagnosis (DCP25 OR: 2.80, 95% CI: 1.08, 7.27; DCP24 OR: 1.95, 95% CI: 0.94, 4.06). Increased odds of previous ovarian cancer diagnosis were also observed with an IQR increase in BPA (OR: 1.93, 95% CI: 1.11, 3.35), and marginally (0.5 ≤ p < 0.1) with an IQR increase in BP3 (OR: 1.76, 95% CI: 1.00, 3.09). Reduced odds of previous uterine cancer were associated with EPB (OR: 0.31, 95% CI: 0.12, 0.85). Finally, odds of previous melanoma diagnosis among women was associated with an IQR increase in BP3 (OR: 1.81, 95% CI: 1.10, 2.96), DCP25 (OR: 2.41, 95% CI: 1.22, 4.76), and DCP24 (OR: 1.85, 95% CI: 1.05, 3.26).

**Fig. 4:**
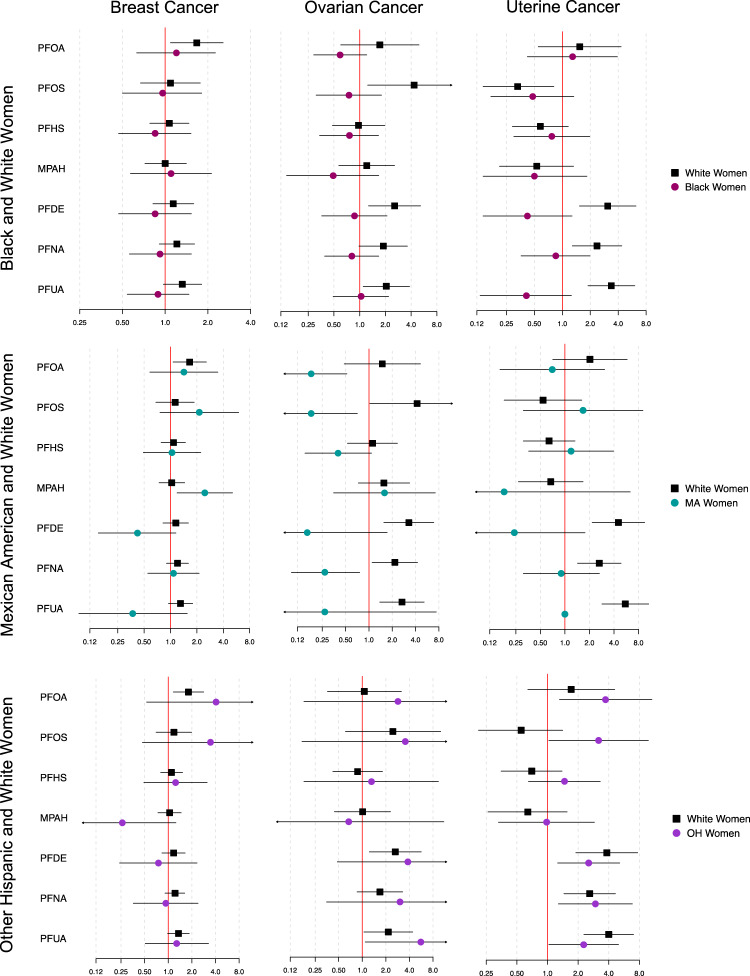
Differential associations between PFAS exposures and previous cancer diagnosis by race among women. Complete corresponding numerical data can be found in the supplementary materials “Supplemental_Tables 8–11.xlsx”. Forest plot reports the odds of each cancer outcome and 95% confidence interval with an IQR increase in PFAS chemical for each race. For each respective row of plots, estimates were generated utilizing subsets of data containing only white women and women of the specified race.

**Fig. 5:**
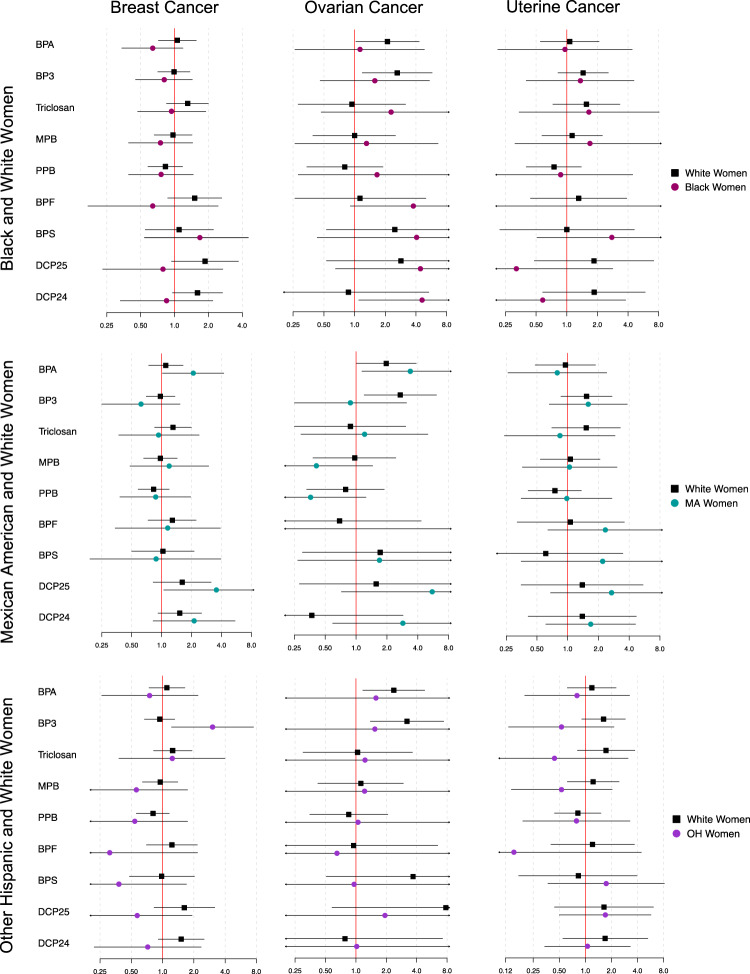
Differential associations between phenol/paraben exposures and previous cancer diagnosis by race among women. Complete corresponding numerical data can be found in the supplementary materials “Supplemental_Tables 8–11.xlsx”. Forest plot reports the odds of each cancer outcome and 95% confidence interval with an IQR increase in phenol/paraben for each race. For each respective row of plots, estimates were generated utilizing subsets of data containing only white women and women of the specified race.

Distribution of cancer outcomes by race are reported in Supplemental Table 7, and differential associations between current exposure biomarkers and previous cancer diagnoses by racial groups are shown in Figs. 4, 5 (complete numerical data is shown in the “Supplemental_Tables 8–11” excel document). There was a greater association between previous prostate cancer diagnosis and an IQR increase in PFNA exposure (p-int=0.043) among other Hispanic men (OR: 2.24, 95% CI: 0.95, 5.29) when compared to White men (OR: 0.89, 95% CI: 0.65, 1.21). Associations between numerous PFAS chemicals and previous ovarian cancer were also modified by race. White women were more likely than Black women to have a previous ovarian cancer diagnosis with an IQR increase in PFOS (OR in White women: 4.34, 95% CI: 1.24, 15.1; OR in Black women: 0.75, 95% CI: 0.31, 1.80; p-int = 0.010) and PFDE (OR in White women: 2.56, 95% CI: 1.27, 5.16; OR in Black women: 0.87, 95% CI: 0.36, 2.09; p-int=0.051), and also more likely to have a previous diagnosis of uterine cancer with IQR increases in PFDE (OR in White women: 3.08, 95% CI: 1.53, 6.21; OR in Black women: 0.42, 95% CI: 0.14, 1.27; p-int=0.002), PFNA (OR in White women: 2.36, 95% CI: 1.28, 4.37; OR in Black women: 0.85, 95% CI: 0.36, 1.98; p-int=0.043), and PFUA (OR in White women: 3.37, 95% CI: 1.89, 6.04; OR in Black women: 0.41, 95% CI: 0.13, 1.25; p-int=0.001). White women were also more likely than Mexican American women to have a previous ovarian cancer diagnosis with an IQR increase in PFOA (p-int=0.007), PFOS (p-int=0.001), PFDE (p-int=0.015), and PFNA (p-int=0.001), and to have a previous uterine cancer diagnosis with an IQR increase in PFDE (p-int=0.006), but low ovarian and uterine cancer case numbers among Mexican American women may have contributed to unreliable effect estimates. Conversely, Mexican American women were more likely than White women to have a previous breast cancer diagnosis with an IQR increase in MPAH (OR in Mexican American women: 2.46, 95% CI: 1.19, 5.09; OR in White women: 1.03, 95% CI: 0.74, 1.45; p-int=0.026), and other Hispanic women were more likely to have a previous uterine cancer diagnosis than White women (OR in other Hispanic women: 3.18, 95% CI: 1.03, 9.82; OR in White women: 0.55, 95% CI: 0.21, 1.41; p-int=0.009). Most associations between previous cancer diagnosis and phenol/paraben exposures did not differ by race among men, but White men were more likely than Black men to have a previous prostate cancer diagnosis with an IQR increase in BP3 (OR in White men: 1.42, 95% CI: 1.07, 1.89; OR in Black men: 0.70, 95% CI: 0.41, 1.21; p-int=0.022) and BPF (OR in White men: 1.40, 95% CI: 0.78, 2.54; OR in Black men: 0.33, 95% CI: 0.10, 1.12; p-int=0.034). Finally, other Hispanic women were more likely than White women to have a previous breast cancer diagnosis with an IQR increase in BP3 (OR in other Hispanic women: 3.03, 95% CI: 1.22, 7.50; OR in White women: 0.94, 95% CI: 0.67, 1.31; p-int=0.017).

## Discussion

Here we have reported numerous associations between current concentrations of biomarkers of exposure to PFAS, phenol, and paraben chemicals and previous cancer diagnoses over multiple NHANES cycles. Of note, the PFAS chemicals PFDE, PFNA, and PFUA were associated with increased odds of prior melanoma diagnosis among women, but not men. Also, among women, concentrations of BPA, BP3, and two dichlorophenols were associated with greater odds of ovarian cancer. Both dichlorophenols showed positive associations with the odds of every cancer type assessed, particularly among women. Finally, greater odds of previous cancer diagnoses among White women were observed with higher PFAS exposure, while Black and Mexican American women were more likely to have a previous cancer diagnosis with increased phenol/paraben exposure.

Numerous epidemiology studies have investigated potential associations between PFAS exposure and melanoma, but no notable effects have been found and most studies assessed only PFOA and PFOS exposures. Importantly, melanoma is the fifth most common cancer in the U.S. and recent estimates indicate increasing incidence in higher-income countries [19]. While the proportion of melanoma diagnoses is higher among White individuals, survival rates have been shown to be significantly lower among individuals who are Black, Hispanic, Asian American, Native American, and Pacific Islander [20]. Two large scale cohort studies have shown a null association between PFOA exposure and melanoma [21, 22], but both studies estimated exposure using indirect modeling rather than biomarker measurements, potentially leading to exposure misclassification and inability to account for inherent biological differences between participants such as PFAS elimination. Previous occupational exposure studies have also reported null associations between PFOA/PFOS and malignant melanoma, but these studies report low melanoma case numbers and are composed of mostly men (>80% male) [23–25]. One occupational study observed increased odds of melanoma with higher exposure to PFOS, but that cohort included only 5 cases of melanoma, reducing the reliability of their results [26]. Importantly, these occupational cohort studies utilized job-exposure matrices to ascertain PFAS exposure levels and thus their results are highly susceptible to exposure misclassification. Though the cohort study design is preferable to cross-sectional studies, the lack of biomonitoring data on the study participants presents a significant limitation to these studies. Further investigation of prospective associations is needed, especially in women based on findings from our study that phenols (DCP25, DCP24, BP3) and PFAS (PFDE, PFNA, and PFUA) were positively associated with previous melanoma diagnosis.

Sex-specific associations between PFAS chemicals and previous melanoma diagnosis, suggest that sex-mediated mechanisms may be at play. Previous work has shown that women are more likely than men to be diagnosed with melanoma, but metastasis and mortality rates are higher among men than women [27]. Differences in melanoma outcomes based on sex may be driven by sex-specific differences in perturbations to biological processes such as cellular immortality, inflammation, oxidative stress, and hormone disruption, which are mechanisms that have been shown to be both influenced by chemical exposures and linked to cancer [10]. For example, a previous review highlighted evidence of sex differences in immune homeostasis (e.g., differences in lymphocyte activation), oxidative stress (e.g., differences in anti-oxidant enzyme levels), and sex hormones (e.g., differences in estrogen levels) [28]. The important role of estrogens during human pregnancy can shed light on how estrogens may also be implicated in cancer development and progression. Estrogens are critical for maintenance of pregnancy as they stimulate blood vessel formation in the uterus. Because melanoma tumor cells express estrogen receptors [29], this angiogenic property of estrogens that is so critical during pregnancy may also promote nourishment of malignant melanomas. Further, it is plausible that environmental toxicants which exhibit estrogenic activity could exacerbate this process. Toxicological evidence for estrogenicity of PFAS is mixed: a recent in vitro study demonstrated PFAS interaction with estrogen receptor-α [30]; another recent study used in vitro and in silico methods to show that particular interactions with the estrogen receptor surface can result in PFAS exerting both estrogenic and antiestrogenic activities [31]; and other recent studies have shown no effect on estrogen levels with PFAS exposure [32, 33]. Clearly, the sexually dimorphic nature of melanoma warrants further investigation in future prospective studies, both in terms of baseline level risk between men and women and the ability of estrogenic environmental insults to further increase risk.

Based on data collected from 1999 to 2015, uterine cancer was one of few cancers increasing in incidence and mortality in the United States [34]. Uterine cancer is the fourth most commonly diagnosed cancer among U.S. women, and previous work has shown racial disparities in both incidence and histological types, with White women showing higher incidence rates than other racial/ethnic groups, and Black women showing higher mortality than other racial/ethnic groups due to diagnoses at more advanced stages of disease [35]. In our study, we observed numerous differences between racial groups, with most associations being significant and positive in White women compared to other racial groups. Notably, increased exposure to PFOA and PFOS was associated with significantly greater odds of previous uterine cancer diagnosis among Other Hispanic women relative to White women, while increased exposure to PFDE, PFNA, and PFUA were significantly associated with greater odds of previous uterine cancer diagnosis among both White and Other Hispanic women. It has been established that elevated circulating estrogens and greater rates of obesity are strongly linked to risk of uterine cancer [36]. Though the links between these factors and endocrine-disrupting chemicals including PFAS and phenols/parabens remain controversial, these may represent modifiable risk factors for targeted intervention strategies.

We observed an inverse association between PFOS exposure and odds of previous thyroid cancer diagnosis among women, while all other associations with thyroid cancer were null. To our knowledge, only one previous study has explored associations between PFAS exposure and risk of thyroid cancer. That community-based analysis found that those living in an area known to have experienced PFAS contamination of drinking water showed greater risk ratios of thyroid cancer relative to those living in unexposed control areas [37]. Additionally, one previous biomonitoring study found that increased urinary levels of the parabens MPB, EPB, and PPB were positively associated with odds of thyroid cancer [38]. Importantly, both previous studies assessed thyroid cancer outcomes among both men and women combined, while our analysis was only able to assess previous thyroid cancer diagnoses in women due to low case numbers among men. Future work should aim to better characterize endocrine disruptor associations with thyroid cancer by disentangling associations between men and women, and by using biomonitoring exposure assessment methods.

Ovarian cancer is the leading cause of death among gynecological cancers and is the seventh most commonly diagnosed cancer among women around the world [39]. Black women are disproportionately affected and have higher odds of more aggressive tumor stages [40]. Ovarian cancer, despite being less common than other cancer types among women, has a low 5-year survival rate due to the advanced stage at which it is usually diagnosed; about 75% of women are diagnosed in late-stage disease and face a 5-year survival rate of about 29% [41]. Current clinical researchers are putting considerable effort into identifying effective screening strategies but there have been no approved protocols to-date [42]. Clearly, identifying environmental exposures that puts one at greater risk of developing ovarian cancer is critical for furthering screening and prevention efforts.

Our results suggest that various environmental chemicals (PFUA, BPA, BP3, and DCP25) are associated with previous diagnosis of ovarian cancer among all women. Previous work has shown that exposure to environmental toxicants can influence cells to undergo the epithelial-mesenchymal transition (EMT), a process, defined by epithelial cells losing their cell-to-cell adhesion properties and becoming migratory, that may be crucial in the transformation of benign cells into malignant cells [43]. Upon treating ovarian cancer cells with BPA, a previous study observed that mRNA expression and protein levels of vimentin and snail, two protein families involved in the EMT, were increased. Further, protein levels of E-cadherin, a cell adhesion protein, were decreased following treatment with BPA [44]. Similarly, another study utilizing a different type of ovarian cancer cell line found that treatment of cells with BPA resulted in stimulated cell migration via upregulation of matrix metalloproteinases and N-cadherin [45]. Ovarian cancer is known to be hormonally driven; about 50% of ovarian cancer cells in humans express higher levels of estrogen receptor-α and -β relative to cells from a normal ovary or benign tumor cells [46]. Accordingly, both previous studies used treatment with estradiol as a positive control and observed that the effects of BPA treatment were similar to that of estradiol, indicating the importance of mitigating exposures to estrogenic chemicals for ovarian cancer prevention.

A previous review illustrates that BPA can regulate the expression of genes in ovarian cancer cells which act on pathways implicated in many of the key characteristics of cancer. For example, genes involved in cell proliferation can be upregulated by BPA, while other genes involved in apoptosis can be downregulated by BPA [47]. However, despite the large number of studies that have implicated BPA in the initiation and/or progression of ovarian cancer, no epidemiology studies to our knowledge have evaluated associations between BPA exposure, or any other phenols, and ovarian cancer. This highlights a significant gap in the environmental epidemiology literature and presents an opportunity to explore impactful mechanisms by which environmental estrogenic compounds may contribute to onset and progression of ovarian cancer.

It is critical for future studies to understand the effects of endocrine active compound exposures on survivors of hormonally-driven cancers. Our findings highlight that across multiple tumor types, individuals with a prior cancer diagnosis have elevated body burdens of a range of toxicants. Hormonally-driven cancers are often treated with hormone therapy to reduce or alter the circulating concentrations of hormones [48]. Exposure to endocrine active compounds could subvert the effects of these therapies and cause disease progression and recurrence [49]. For example, approximately 70% of breast cancers express the estrogen receptor [50]. These breast cancers are often treated with antiestrogen therapies. Unexpected exposure to estrogenic xenobiotic compounds could promote the growth and spread of estrogen receptor-positive tumors. This potential impact on long term cancer patient outcomes is particularly salient in light of the high rates of distant recurrences in estrogen-positive breast cancer survivors up to 20 years following the cessation of treatment [51]. Our findings build upon a growing literature showing that cancer survivors are an important population for endocrine active chemical biomonitoring and interventions.

We observed that various associations between environmental chemical exposures and previous cancer diagnoses were modified by race. Environmental exposures may differ by racial groups through various sources. For example, chemicals such as phthalates, phenols, and parabens may be found at higher concentrations in certain beauty products (e.g., hair straightening chemicals and skin-lightening creams) that are marketed to Black, Asian, and Latina women [52]. Another example is evidenced by disparities in PFAS water contamination, with a recent report from the Natural Resources Defense Council indicating that many counties in California with higher CalEnviroScreen scores (indicating greater pollution and socioeconomic disadvantage) also had higher detected PFAS in drinking water systems [53]. A previous study in NHANES showed that significant racial disparities exist in biomonitored environmental toxicants, including several exposures included in this analysis [16]. Of note, the aforementioned study observed that non-White racial groups had much higher concentrations of chemical biomarkers including DCP25, MPB, PPB, BPS, and PFDA, while White participants had higher levels of other chemicals including BPF, BP3, PFOA, and PFOS. These exposure levels partly contextualize our findings that White men had higher odds of previous prostate cancer with elevated exposures to BP3 and BPF, and White women had higher odds of previous ovarian cancer with elevated exposures to PFOS and PFOA. Additionally, despite greater increases of uterine cancer incidence among Black women compared to White women [54], we observed that White women were more likely to have previous uterine cancer with increasing biomarker levels of PFDE, PFNA, and PFUA. Interestingly, Nguyen and colleagues showed that Black women had higher concentrations of PFNA than White women, suggesting that the positive association between PFNA and previous uterine cancer among White women compared to Black women may not be influenced by trends in incidence or exposure levels to PFNA between racial groups. Finally, we observed that Mexican-American women had higher odds of previous breast cancer with elevated exposure to MPAH, and other Hispanic women had higher odds of previous breast cancer with elevated exposure to BP3. Accordingly, a previous review article demonstrates that Hispanic women are at greater risk of breast cancer-specific mortality when compared to non-Hispanic White women [55]. There may also be underlying metabolic factors influencing the relationship between endocrine-disrupting chemicals and cancer risk. For example, a previous multi-omics investigation identified differences in genetic and epigenetic loci relevant for xenobiotic metabolism based on genetic ancestry, which may be relevant since endocrine-disrupting chemicals are metabolized by overlapping enzymes, including cytochrome p450 [56]. Future prospective studies should not only consider disparities in exposure and cancer risks, but also evaluate potential sources of environmental contamination to endocrine-disrupting chemicals to guide potential interventions.

These results highlight the need to carefully consider the use of survey regression methods based on whether the study hypothesis is aimed at obtaining results that are generalizable or specific to vulnerable populations. NHANES oversamples racial/ethnic minorities, which can be very useful when trying to evaluate rare disease states as outcomes in those populations. However, when survey regression methods are applied, the results generated are targeted at being generalizable to the entire United States population rather than being truly representative of the study population, which has the desired larger population of minority groups. Thus, if an association is observed among a minority group but null among non-Hispanic White participants, the survey regression results will be influenced towards the null to account for the oversampling of the minority group. We observed this in our analysis with PFDE exposure and odds of previous ovarian cancer. Standard regression analysis showed a non-significant positive association, but sensitivity analyses revealed that the association was observed only among non-Hispanic White participants. Accordingly, survey regression methods also resulted in a positive association. Thus, survey regression methods will generate more generalizable results, but if there are true differences in associations between racial groups, the survey regression results will be more representative of non-Hispanic White participants than of the minority groups.

This analysis was subject to various limitations. First, our outcomes were previous cancer diagnoses and therefore causality cannot be determined. While we would have liked to account for the time between cancer diagnoses and biomarker measurement, this information was not available in NHANES. Further, because our exposures were measured after the cancer diagnoses occurred, reverse causation is a possibility if behavioral changes occurred. Subsequent treatment for cancer may also influence concentrations of endocrine-disrupting chemicals through altered metabolism, which may also be an important source of exposure misclassification among those with previous cancer diagnoses. Additionally, we have assumed that exposure biomarker measurements are accurate proxies of historical exposure levels, and so there is high risk for exposure misclassification. Despite this limitation, there is still utility in assessing PFAS and phenol/paraben profiles among previous cancer patients to inform prospective hypotheses in emerging cohort studies. Additionally, our results were likely subject to bias from unmeasured confounding factors such as family history of cancer or anatomical alterations such as ovariectomy and thyroidectomy. Our regression models may not have accounted for any correlations between covariates and biomarkers, possibly resulting in inflated associations. Similarly, we did not set multiple comparison thresholds, therefore some associations may be false positives. However, future prospective studies can build on our preliminary findings to perform targeted hypothesis testing on specific environmental contaminants. Another limitation includes potential outcome misclassification since previous cancer diagnosis was assessed using self-report questionnaire data. A previous study identified the validity of self-reported cancers with data from state cancer registries and while they identified fairly good accuracy (sensitivity of ≥ 0.9) for certain cancers (e.g., breast and prostate) [57], future studies should build on our preliminary findings using gold-standard cancer diagnosis for outcome phenotyping.

This study was also strong in a number of ways. Compared to previous studies, we leveraged NHANES data to investigate multiple classes of endocrine-disrupting chemicals to inform prioritization and hypothesis-driven investigation of environmental exposures in future prospective study designs. Additionally, our approach helps build the foundation for supervised multi-pollutant chemical mixtures analyses that intend to delineate chemical class-specific effects and potential interactions between chemicals. Multi-chemical class exposure assessment is becoming more common with technological advancements in high-throughput assays, however, these can be cost-prohibitive and time-consuming in certain contexts with limited resources. We also contribute toward reporting exploratory associations with understudied cancers in the context of endocrine-disrupting chemicals. For example, this is the first epidemiological study to assess phenols exposure in the context of previous ovarian cancer diagnosis. Further, this is the first NHANES analysis to investigate racial/ethnic disparities in associations between environmental exposures and previous cancer diagnoses. We also add to the current body of literature suggesting a role for estrogens in the onset and progression of ovarian cancer and melanoma, which could help inform future mechanistic and experimental studies as well as risk assessment and prevention efforts.

In conclusion, we report various associations between exposure to environmental chemicals and previous cancer diagnoses that have not been previously explored. Several PFAS chemicals were positively associated with odds of previous melanoma diagnosis among only women, and various PFAS and phenols were positively associated with odds of previous ovarian cancer diagnosis. These findings highlight a sexually dimorphic nature of melanoma risk, as well as a potential estrogen-dependent mechanism for both cancer types. We also showed differential associations between environmental exposures and previous cancer diagnoses by racial groups, underscoring racial disparities that exist both in innate risk of cancer outcomes and in exposures to environmental toxicants. Future work in prospective cancer studies should aim to explore the roles of estrogenic chemicals and estrogen disruption in the pathology of melanoma and ovarian cancer and consider racial disparities when evaluating cancer mechanisms and risk. Findings from this study can be used to help inform and prioritize toxicants for policies surrounding greater surveillance of chemical exposures and risk assessment in communities with existing or emerging risk of environmental contamination.

### Supplementary Information

Reproducibility Checklist

Supplemental_Tables1-7

Supplemental_Tables8–11

## Data Availability

NHANES data is publicly available. The analytical dataset for this study and code can be available upon request (Amber Cathey [acathey@umich.edu], Max Aung [maxaung@usc.edu]).
