# Supplementary material for: Exploratory profiles of phenols, parabens, and per- and poly-fluoroalkyl substances among NHANES study participants in association with previous cancer diagnoses
Source: J Expo Sci Environ Epidemiol. 2023 Sep 18;33(5):687–98. doi: 10.1038/s41370-023-00601-6 (PMC10541322; doi:10.1038/s41370-023-00601-6)
Supplement: Supplementary file 2 — Supplemental_Tables1-7 [file 41370_2023_601_MOESM2_ESM.docx]

**Supplemental Table 1**. Distributions of PFAS chemicals from NHANES 2005-2018 participants aged 20 years and older who provided complete data on selected covariates and who gave cancer outcome data.

| **PFAS (ng/mL)** |  |  |  |  |  |  |  |  |  |  |  |
| --- | --- | --- | --- | --- | --- | --- | --- | --- | --- | --- | --- |
| **Men (N=6360)** | **N** | **N>LOD** | **Min** | **P25** | **P50** | **P75** | **P90** | **Max** | **GM** | **GSD** | **IQR** |
| **PFOA** | 4900 | 4874 | 0.07 | 1.50 | 2.50 | 4.40 | 6.70 | 104 | 2.48 | 2.25 | 2.90 |
| **PFOS** | 4900 | 4885 | 0.07 | 3.80 | 8.20 | 17.2 | 29.6 | 281 | 8.06 | 2.87 | 13.4 |
| **PFHS** | 6360 | 6287 | 0.07 | 1.20 | 1.90 | 3.00 | 4.80 | 81.6 | 1.85 | 2.31 | 1.80 |
| **MPAH** | 6360 | 3765 | 0.06 | 0.07 | 0.12 | 0.30 | 0.70 | 11.9 | 0.17 | 2.57 | 0.23 |
| **PFDE** | 6360 | 5293 | 0.07 | 0.14 | 0.20 | 0.40 | 0.70 | 51.3 | 0.24 | 2.28 | 0.26 |
| **PFNA** | 6360 | 6246 | 0.058 | 0.50 | 0.82 | 1.39 | 2.05 | 18.0 | 0.82 | 2.19 | 0.89 |
| **PFUA** | 6360 | 3364 | 0.07 | 0.07 | 0.14 | 0.20 | 0.40 | 77.4 | 0.15 | 2.23 | 0.13 |
| **Women (N=6886)** | **N** | **N>LOD** | **Min** | **P25** | **P50** | **P75** | **P90** | **Max** | **GM** | **GSD** | **IQR** |
| **PFOA** | 5192 | 5167 | 0.07 | 1.00 | 1.90 | 3.30 | 5.20 | 52.8 | 1.78 | 2.43 | 2.30 |
| **PFOS** | 5192 | 5170 | 0.07 | 2.20 | 4.90 | 10.9 | 20.4 | 178 | 4.86 | 3.07 | 8.70 |
| **PFHS** | 6886 | 6771 | 0.07 | 0.50 | 1.00 | 1.80 | 3.30 | 42.7 | 0.99 | 2.62 | 1.30 |
| **MPAH** | 6886 | 3827 | 0.06 | 0.07 | 0.10 | 0.30 | 0.60 | 12.2 | 0.15 | 2.45 | 0.23 |
| **PFDE** | 6886 | 5313 | 0.07 | 0.10 | 0.20 | 0.30 | 0.60 | 14.8 | 0.21 | 2.30 | 0.20 |
| **PFNA** | 6886 | 6700 | 0.058 | 0.40 | 0.70 | 1.15 | 1.80 | 25.7 | 0.67 | 2.36 | 0.75 |
| **PFUA** | 6886 | 3385 | 0.07 | 0.07 | 0.14 | 0.20 | 0.40 | 10.3 | 0.14 | 2.16 | 0.13 |

**Supplemental Table 2**. Distributions of phenol chemicals from NHANES 2005-2016 participants aged 20 years and older who provided complete data on selected covariates and who gave cancer outcome data.

| **Phenols (ng/mL)** |  |  |  |  |  |  |  |  |  |  |  |
| --- | --- | --- | --- | --- | --- | --- | --- | --- | --- | --- | --- |
| **Men (N=3606)** | **N** | **N>LOD** | **Min** | **P25** | **P50** | **P75** | **P90** | **Max** | **GM** | **GSD** | **IQR** |
| **BPA** | 3606 | 3368 | 0.14 | 0.80 | 1.70 | 3.30 | 6.50 | 574 | 1.68 | 2.97 | 2.50 |
| **BP3** | 3606 | 3427 | 0.28 | 3.20 | 9.20 | 35.0 | 156 | 99887 | 11.3 | 7.50 | 31.8 |
| **Triclosan** | 3606 | 2704 | 1.20 | 1.65 | 7.90 | 51.4 | 284 | 3620 | 12.3 | 7.43 | 49.8 |
| **BPB** | 3606 | 772 | 0.07 | 0.07 | 0.14 | 0.14 | 0.60 | 1150 | 0.17 | 3.46 | 0.07 |
| **EPB** | 3606 | 1452 | 0.71 | 0.71 | 0.71 | 2.50 | 12.4 | 1330 | 1.64 | 3.91 | 1.79 |
| **MPB** | 3606 | 3560 | 0.71 | 9.50 | 29.1 | 114 | 384 | 10000 | 34.6 | 5.60 | 105 |
| **PPB** | 3606 | 3326 | 0.07 | 0.60 | 2.20 | 15.2 | 75.0 | 3915 | 3.15 | 8.91 | 14.6 |
| **BPF** | 1406 | 808 | 0.14 | 0.14 | 0.30 | 0.90 | 3.50 | 241 | 0.45 | 4.15 | 0.76 |
| **BPS** | 1406 | 1297 | 0.07 | 0.20 | 0.50 | 1.10 | 2.60 | 140 | 0.54 | 3.58 | 0.90 |
| **Triclocarban** | 1407 | 545 | 0.07 | 0.07 | 0.07 | 0.20 | 4.02 | 588 | 0.19 | 6.51 | 0.13 |
| **DCP25** | 1407 | 1383 | 0.07 | 1.00 | 2.90 | 13.1 | 70.7 | 25965 | 4.30 | 8.58 | 12.1 |
| **DCP24** | 1407 | 1317 | 0.07 | 0.30 | 0.60 | 1.40 | 3.74 | 767 | 0.72 | 4.19 | 1.10 |
| **Women (N=3807)** | **N** | **N>LOD** | **Min** | **P25** | **P50** | **P75** | **P90** | **Max** | **GM** | **GSD** | **IQR** |
| **BPA** | 3807 | 3485 | 0.14 | 0.70 | 1.50 | 3.10 | 6.00 | 965 | 1.49 | 3.12 | 2.40 |
| **BP3** | 3807 | 3672 | 0.28 | 5.20 | 18.5 | 101 | 596 | 23641 | 24.5 | 9.87 | 95.8 |
| **Triclosan** | 3807 | 2901 | 1.20 | 2.00 | 7.80 | 41.6 | 234 | 9572 | 11.7 | 6.99 | 39.6 |
| **BPB** | 3807 | 2057 | 0.07 | 0.10 | 0.20 | 1.80 | 10.1 | 860 | 0.47 | 7.17 | 1.70 |
| **EPB** | 3807 | 2244 | 0.71 | 0.71 | 1.80 | 13.2 | 59.1 | 3130 | 3.56 | 6.29 | 12.4 |
| **MPB** | 3807 | 3787 | 0.71 | 34.2 | 122 | 357 | 822 | 17300 | 105 | 5.37 | 323 |
| **PPB** | 3807 | 3727 | 0.07 | 4.50 | 22.9 | 81.1 | 231 | 7210 | 17.9 | 7.98 | 76.6 |
| **BPF** | 1578 | 869 | 0.14 | 0.14 | 0.20 | 0.80 | 2.90 | 247 | 0.41 | 4.06 | 0.66 |
| **BPS** | 1578 | 1411 | 0.07 | 0.20 | 0.50 | 1.20 | 2.60 | 68 | 0.51 | 3.63 | 1.00 |
| **Triclocarban** | 1579 | 599 | 0.07 | 0.07 | 0.07 | 0.20 | 2.52 | 457 | 0.18 | 5.39 | 0.13 |
| **DCP25** | 1579 | 1536 | 0.07 | 0.90 | 3.00 | 16.2 | 86.6 | 41600 | 4.29 | 9.84 | 15.3 |
| **DCP24** | 1579 | 1468 | 0.07 | 0.30 | 0.60 | 1.50 | 4.60 | 1261 | 0.73 | 4.47 | 1.20 |

**Supplemental Table 3**. Odds of each cancer type with an IQR increase in PFAS chemicals.

| Men | Reproductive Cancers | Prostate Cancer |  |  |  | Melanoma |
| --- | --- | --- | --- | --- | --- | --- |
| PFOA | 0.90 (0.70, 1.15) | 0.88 (0.69, 1.12) |  |  |  | 0.87 (0.54, 1.40) |
| PFOS | 1.00 (0.75, 1.32) | 1.01 (0.76, 1.34) |  |  |  | 0.68 (0.42, 1.10) |
| PFHS | 1.04 (0.88, 1.23) | 1.04 (0.88, 1.23) |  |  |  | 1.02 (0.73, 1.41) |
| MPAH | 0.95 (0.74, 1.21) | 0.92 (0.72, 1.17) |  |  |  | 0.72 (0.44, 1.18) |
| PFDE | 1.04 (0.85, 1.27) | 1.05 (0.85, 1.28) |  |  |  | 0.78 (0.51, 1.18) |
| PFNA | 0.94 (0.75, 1.17) | 0.95 (0.76, 1.18) |  |  |  | 0.75 (0.48, 1.17) |
| PFUA | 0.91 (0.73, 1.12) | 0.94 (0.76, 1.17) |  |  |  | 0.78 (0.47, 1.30) |
| Women | Reproductive Cancers | Breast Cancer | Ovarian Cancer | Uterine Cancer | Thyroid Cancer | Melanoma |
| PFOA | 1.09 (0.85, 1.41) | 1.14 (0.83, 1.56) | 0.68 (0.40, 1.15) | 1.62 (0.94, 2.80) | 0.70 (0.38, 1.30) | 1.70 (0.76, 3.81) |
| PFOS | 0.88 (0.67, 1.16) | 0.90 (0.64, 1.27) | 0.89 (0.45, 1.78) | 0.93 (0.52, 1.67) | 0.53 (0.28, 1.00) | 1.39 (0.51, 3.84) |
| PFHS | 1.00 (0.82, 1.22) | 1.04 (0.81, 1.32) | 0.86 (0.53, 1.39) | 0.96 (0.63, 1.47) | 0.62 (0.36, 1.06) | 1.22 (0.74, 2.04) |
| MPAH | 0.91 (0.72, 1.15) | 1.00 (0.76, 1.31) | 1.01 (0.55, 1.85) | 0.63 (0.34, 1.17) | 1.32 (0.70, 2.51) | 1.08 (0.61, 1.89) |
| PFDE | 0.95 (0.77, 1.18) | 0.83 (0.64, 1.07) | 1.45 (0.86, 2.45) | 1.41 (0.93, 2.15) | 1.44 (0.81, 2.56) | **2.07 (1.25, 3.43)** |
| PFNA | 1.06 (0.88, 1.27) | 0.99 (0.80, 1.24) | 1.13 (0.69, 1.84) | **1.55 (1.03, 2.34)** | 0.93 (0.55, 1.60) | **1.72 (1.09, 2.73)** |
| PFUA | 1.05 (0.87, 1.28) | 0.97 (0.77, 1.23) | **1.61 (1.00, 2.59)** | 1.29 (0.86, 1.94) | 1.12 (0.66, 1.92) | **1.76 (1.07, 2.89)** |

**Supplemental Table 4**. Odds of each cancer type with an IQR increase in each phenol/paraben chemical.

| Men | Reproductive Cancers | Prostate Cancer |  |  |  | Melanoma |
| --- | --- | --- | --- | --- | --- | --- |
| BPA | 0.87 (0.63, 1.19) | 0.80 (0.58, 1.12) |  |  |  | 0.68 (0.33, 1.40) |
| BP3 | 1.12 (0.89, 1.42) | 1.15 (0.90, 1.46) |  |  |  | 1.34 (0.81, 2.21) |
| Triclosan | 1.10 (0.77, 1.56) | 1.14 (0.79, 1.63) |  |  |  | 1.38 (0.64, 2.98) |
| BPB* | 0.91 (0.39, 2.11) | 0.94 (0.40, 2.20) |  |  |  | 0.89 (0.10, 7.51) |
| EPB* | 0.67 (0.34, 1.30) | 0.72 (0.37, 1.41) |  |  |  | 0.55 (0.07, 4.54) |
| MPB | 1.12 (0.82, 1.54) | 1.23 (0.89, 1.70) |  |  |  | 1.34 (0.66, 2.69) |
| PPB | 1.24 (0.92, 1.66) | **1.35 (1.00, 1.83)** |  |  |  | 1.40 (0.71, 2.75) |
| BPF | 1.07 (0.69, 1.67) | 0.93 (0.57, 1.54) |  |  |  | 1.24 (0.53, 2.88) |
| BPS | 1.24 (0.80, 1.93) | 1.28 (0.80, 2.03) |  |  |  | 0.99 (0.37, 2.64) |
| Triclocarban* | 1.05 (0.44, 2.48) | 0.97 (0.39, 2.39) |  |  |  | 0.97 (0.18, 5.33) |
| DCP25 | 1.34 (0.91, 1.99) | 1.29 (0.85, 1.94) |  |  |  | 1.22 (0.58, 2.58) |
| DCP24 | 1.19 (0.84, 1.70) | 1.20 (0.83, 1.74) |  |  |  | 0.81 (0.38, 1.74) |
| Women | Reproductive Cancers | Breast Cancer | Ovarian Cancer | Uterine Cancer | Thyroid Cancer | Melanoma |
| BPA | 1.11 (0.87, 1.42) | 1.06 (0.78, 1.43) | **1.93 (1.11, 3.35)** | 0.87 (0.51, 1.48) | 0.62 (0.20, 1.90) | 0.99 (0.53, 1.83) |
| BP3 | 1.12 (0.91, 1.38) | 1.04 (0.81, 1.34) | **1.76 (1.00, 3.09)** | 1.25 (0.81, 1.92) | 1.43 (0.62, 3.32) | **1.81 (1.10, 2.96)** |
| Triclosan | 1.18 (0.90, 1.55) | 1.16 (0.84, 1.60) | 1.09 (0.49, 2.41) | 1.18 (0.67, 2.08) | 0.17 (0.03, 1.12) | 0.95 (0.49, 1.86) |
| BPB* | 0.85 (0.57, 1.27) | 0.96 (0.60, 1.54) | 0.56 (0.17, 1.85) | 0.69 (0.30, 1.57) | 1.58 (0.72, 3.46) | 1.10 (0.40, 3.02) |
| EPB* | 0.70 (0.47, 1.05) | 0.87 (0.54, 1.40) | 0.73 (0.24, 2.24) | **0.31 (0.12, 0.85)** | 0.60 (0.17, 2.15) | 2.33 (0.87, 6.24) |
| MPB | 0.92 (0.72, 1.17) | 0.96 (0.71, 1.30) | 0.80 (0.41, 1.56) | 0.95 (0.58, 1.54) | 0.93 (0.32, 2.69) | 1.71 (0.90, 3.24) |
| PPB | 0.79 (0.63, 1.00) | 0.84 (0.64, 1.12) | 0.73 (0.38, 1.39) | 0.76 (0.48, 1.21) | 1.03 (0.38, 2.76) | 1.58 (0.88, 2.86) |
| BPF | 1.05 (0.74, 1.50) | 1.09 (0.70, 1.68) | 1.16 (0.49, 2.73) | 0.91 (0.42, 1.99) | 0.27 (0.04, 1.67) | 1.40 (0.74, 2.67) |
| BPS | 1.08 (0.71, 1.65) | 0.92 (0.54, 1.54) | 2.23 (0.74, 6.72) | 1.38 (0.59, 3.24) | 1.65 (0.49, 5.54) | 1.16 (0.51, 2.65) |
| Triclocarban* | 0.88 (0.43, 1.83) | 0.67 (0.24, 1.87) | 0.77 (0.13, 4.59) | 1.17 (0.33, 4.15) | 1.23 (0.83, 1.82) | 0.37 (0.05, 2.96) |
| DCP25 | **1.61 (1.13, 2.29)** | 1.49 (0.95, 2.34) | **2.80 (1.08, 7.27)** | 1.49 (0.75, 2.93) | 2.38 (0.85, 6.65) | **2.41 (1.22, 4.76)** |
| DCP24 | **1.42 (1.06, 1.90)** | 1.36 (0.94, 1.95) | 1.95 (0.94, 4.06) | 1.20 (0.68, 2.14) | 1.56 (0.68, 3.58) | **1.85 (1.05, 3.26)** |

*Exposures modeled categorically with all concentrations below the LOD being the reference category and effect estimates representing an increase to concentrations at or above the median among those above the LOD.

**Supplemental Table 5**. Survey regression results showing the odds of each cancer type with an IQR increase in PFAS chemicals.

| Men | All Reproductive Cancers | Prostate Cancer |  |  |  | Melanoma |
| --- | --- | --- | --- | --- | --- | --- |
| PFOA | 0.71 (0.47, 1.07) | 0.70 (0.45, 1.07) |  |  |  | 0.83 (0.46, 1.50) |
| PFOS | 0.83 (0.52, 1.32) | 0.92 (0.59, 1.44) |  |  |  | 0.60 (0.31, 1.19) |
| PFHS | 1.00 (0.72, 1.39) | 0.96 (0.69, 1.33) |  |  |  | 1.01 (0.61, 1.68) |
| MPAH | 1.02 (0.67, 1.54) | 1.01 (0.71, 1.43) |  |  |  | 0.69 (0.39, 1.21) |
| PFDE | 1.01 (0.68, 1.49) | 1.16 (0.82, 1.64) |  |  |  | 0.57 (0.25, 1.31) |
| PFNA | 0.90 (0.61, 1.33) | 0.97 (0.63, 1.47) |  |  |  | 0.74 (0.41, 1.33) |
| PFUA | 0.96 (0.64, 1.45) | 1.20 (0.85, 1.70) |  |  |  | 0.62 (0.29, 1.29) |
| Women | All Reproductive Cancers | Breast Cancer | Ovarian Cancer | Uterine Cancer | Thyroid Cancer | Melanoma |
| PFOA | 1.22 (0.77, 1.91) | 1.15 (0.69, 1.91) | 1.12 (0.48, 2.60) | 1.67 (0.29, 9.75) | 1.51 (0.31, 7.31) | 1.24 (0.59, 2.64) |
| PFOS | 0.91 (0.58, 1.43) | 0.95 (0.65, 1.39) | 1.87 (0.74, 4.77) | 0.60 (0.13, 2.68) | 0.93 (0.27, 3.22) | 0.74 (0.26, 2.10) |
| PFHS | 1.08 (0.78, 1.50) | 1.15 (0.84, 1.57) | 0.97 (0.48, 1.97) | 0.86 (0.33, 2.28) | 0.67 (0.35, 1.30) | 1.10 (0.68, 1.79) |
| MPAH | 0.82 (0.60, 1.13) | 0.87 (0.60, 1.28) | 1.10 (0.50, 2.45) | **0.45 (0.21, 0.93)** | 1.79 (0.60, 5.34) | 0.57 (0.27, 1.20) |
| PFDE | 1.20 (0.90, 1.60) | 0.98 (0.65, 1.49) | **1.93 (1.33, 2.80)** | 2.01 (0.76, 5.34) | 1.53 (0.94, 2.50) | 1.50 (0.65, 3.50) |
| PFNA | 1.21 (0.84, 1.74) | 1.08 (0.73, 1.59) | 1.50 (0.95, 2.39) | 1.88 (0.52, 6.83) | 1.41 (0.87, 2.30) | 1.39 (0.58, 3.34) |
| PFUA | **1.45 (1.06, 1.98)** | 1.28 (0.84, 1.95) | **2.14 (1.12, 4.11)** | 2.20 (0.97, 4.97) | 1.46 (0.91, 2.35) | 1.41 (0.49, 4.06) |

*Exposures modeled categorically with all concentrations below the LOD being the reference category and effect estimates representing an increase to concentrations at or above the median among those above the LOD.

**Supplemental Table 6**. Survey regression results showing the odds of each cancer type with an IQR increase in each phenol/paraben chemical.

| Men | All Reproductive Cancers | Prostate Cancer |  |  |  | Melanoma |
| --- | --- | --- | --- | --- | --- | --- |
| BPA | 1.11 (0.79, 1.55) | 0.92 (0.65, 1.31) |  |  |  | 0.88 (0.40, 1.95) |
| BP3 | 1.21 (0.88, 1.66) | 1.32 (0.99, 1.75) |  |  |  | **1.79 (1.09, 2.92)** |
| Triclosan | 0.83 (0.50, 1.37) | 1.03 (0.63, 1.70) |  |  |  | 1.25 (0.50, 3.10) |
| BPB* | 1.02 (0.88, 1.19) | 1.07 (0.94, 1.21) |  |  |  | 0.80 (0.50, 1.29) |
| EPB* | 0.81 (0.68, 0.96) | 0.84 (0.70, 1.02) |  |  |  | 0.96 (0.69, 1.34) |
| MPB | 0.90 (0.62, 1.29) | 1.10 (0.73, 1.67) |  |  |  | 1.19 (0.47, 3.04) |
| PPB | 1.03 (0.74, 1.44) | 1.24 (0.87, 1.78) |  |  |  | 1.34 (0.66, 2.73) |
| BPF | 1.22 (0.77, 1.93) | 0.95 (0.53, 1.71) |  |  |  | 0.99 (0.28, 3.54) |
| BPS | 1.20 (0.75, 1.93) | 1.26 (0.70, 2.29) |  |  |  | 0.84 (0.36, 1.94) |
| Triclocarban* | 0.97 (0.86, 1.09) | 0.98 (0.83, 1.15) |  |  |  | 0.88 (0.62, 1.24) |
| DCP25 | 1.56 (0.96, 2.53) | 1.29 (0.82, 2.01) |  |  |  | 0.96 (0.61, 1.52) |
| DCP24 | 1.03 (0.61, 1.74) | 1.03 (0.56, 1.90) |  |  |  | 0.83 (0.45, 1.54) |
| Women | All Reproductive Cancers | Breast Cancer | Ovarian Cancer | Uterine Cancer | Thyroid Cancer | Melanoma |
| BPA | 1.02 (0.74, 1.41) | 0.96 (0.62, 1.48) | 1.19 (0.35, 4.09) | 0.89 (0.52, 1.52) | 0.60 (0.14, 2.64) | 0.93 (0.54, 1.60) |
| BP3 | 1.12 (0.90, 1.39) | 1.04 (0.78, 1.38) | 2.64 (1.53, 4.54) | 1.21 (0.75, 1.93) | 1.40 (0.81, 2.43) | 2.23 (1.30, 3.80) |
| Triclosan | **1.33 (1.02, 1.74)** | 1.28 (0.94, 1.72) | 0.72 (0.21, 2.46) | 1.54 (0.86, 2.75) | 0.15 (0.02, 1.44) | 0.98 (0.48, 2.00) |
| BPB* | 1.08 (0.78, 1.49) | 1.06 (0.71, 1.58) | 0.71 (0.26, 1.96) | 1.14 (0.66, 1.97) | 1.15 (0.49, 2.70) | 0.86 (0.50, 1.48) |
| EPB* | 0.80 (0.57, 1.13) | 0.89 (0.58, 1.35) | 0.68 (0.18, 2.51) | 0.50 (0.20, 1.25) | 0.74 (0.28, 1.99) | 1.34 (0.68, 2.66) |
| MPB | 1.02 (0.72, 1.43) | 0.97 (0.63, 1.49) | 0.82 (0.44, 1.53) | 1.29 (0.82, 2.04) | 1.05 (0.32, 3.45) | 1.65 (1.06, 2.55) |
| PPB | 0.81 (0.60, 1.11) | 0.76 (0.52, 1.13) | 0.71 (0.30, 1.66) | 1.01 (0.70, 1.45) | 1.20 (0.53, 2.73) | 1.36 (0.84, 2.22) |
| BPF | 1.05 (0.71, 1.55) | 1.22 (0.81, 1.82) | 0.62 (0.17, 2.32) | 0.78 (0.22, 2.79) | 0.12 (0.01, 1.37) | 1.64 (1.13, 2.38) |
| BPS | 1.07 (0.67, 1.71) | 0.95 (0.50, 1.83) | 2.52 (1.45, 4.36) | 1.25 (0.87, 1.78) | 1.76 (0.85, 3.65) | 0.84 (0.40, 1.79) |
| Triclocarban* | 0.85 (0.70, 1.05) | 0.84 (0.68, 1.03) | 0.76 (0.53, 1.09) | 0.92 (0.56, 1.50) | 0.99 (0.65, 1.51) | 0.78 (0.43, 1.43) |
| DCP25 | 1.66 (0.99, 2.77) | 1.58 (0.87, 2.85) | 2.57 (1.56, 4.24) | 1.63 (0.61, 4.39) | 1.74 (0.62, 4.93) | 3.30 (1.57, 6.93) |
| DCP24 | 1.32 (0.86, 2.02) | 1.29 (0.77, 2.15) | 0.96 (0.17, 5.51) | 1.25 (0.47, 3.27) | 0.79 (0.18, 3.44) | 2.01 (1.01, 4.00) |

*Exposures modeled categorically with all concentrations below the LOD being the reference category and effect estimates representing an increase to concentrations at or above the median among those above the LOD.

**Supplemental Table 7**. Distributions of cancer outcomes by race in the PFAS and phenols datasets.

| PFAS Dataset |  | **Men** | | | **Women** | | | | | |
| --- | --- | --- | --- | --- | --- | --- | --- | --- | --- | --- |
|  |  | **Repro. Cancers** | **Prostate Cancer** | **Melanoma** | **Repro. Cancers** | **Breast Cancer** | **Ovarian Cancer** | **Uterine Cancer** | **Thyroid Cancer** | **Melanoma** |
| Non-Hispanic White | No | 3212 | 3221 | 3296 | 3273 | 3305 | 3403 | 2923 | 3411 | 3389 |
|  | Yes | 143 | 134 | 59 | 153 | 121 | 23 | 17 | 15 | 37 |
| Non-Hispanic Black | No | 1625 | 1625 | 1693 | 1817 | 1836 | 1856 | 1759 | 1858 | 1867 |
|  | Yes | 68 | 68 | 0 | 50 | 31 | 11 | 10 | 9 | 0 |
| Mexican American | No | 1200 | 1200 | 1210 | 1330 | 1346 | 1365 | 1297 | 1368 | 1368 |
|  | Yes | 12 | 12 | 2 | 40 | 24 | 5 | 11 | 2 | 2 |
| Other Hispanic | No | 711 | 711 | 728 | 885 | 904 | 914 | 846 | 915 | 917 |
|  | Yes | 19 | 19 | 2 | 35 | 16 | 6 | 16 | 5 | 3 |
| Other | No | 998 | 998 | 1014 | 1066 | 1074 | 1097 | 1040 | 1093 | 1096 |
|  | Yes | 18 | 18 | 2 | 31 | 23 | 0 | 9 | 4 | 1 |
| Phenols Dataset |  | **Men** | | | **Women** | | | | | |
|  |  | **Repro. Cancers** | **Prostate Cancer** | **Melanoma** | **Repro. Cancers** | **Breast Cancer** | **Ovarian Cancer** | **Uterine Cancer** | **Thyroid Cancer** | **Melanoma** |
| Non-Hispanic White | No | 2109 | 2116 | 2174 | 2117 | 2148 | 2217 | 1913 | 2224 | 2204 |
|  | Yes | 92 | 85 | 27 | 113 | 82 | 13 | 22 | 6 | 26 |
| Non-Hispanic Black | No | 1063 | 1063 | 1106 | 1133 | 1144 | 1170 | 1106 | 1174 | 1177 |
|  | Yes | 43 | 43 | 0 | 44 | 33 | 7 | 4 | 3 | 0 |
| Mexican American | No | 784 | 784 | 789 | 812 | 827 | 838 | 790 | 843 | 843 |
|  | Yes | 6 | 6 | 1 | 32 | 17 | 6 | 9 | 1 | 1 |
| Other Hispanic | No | 435 | 435 | 446 | 527 | 534 | 546 | 513 | 546 | 546 |
|  | Yes | 12 | 12 | 1 | 20 | 13 | 1 | 7 | 1 | 1 |
| Other | No | 523 | 524 | 531 | 529 | 532 | 540 | 517 | 540 | 541 |
|  | Yes | 9 | 8 | 1 | 12 | 9 | 1 | 2 | 1 | 0 |
